# Quantitative Integration of FRET and Molecular Dynamics for Modeling Flexible Peptides

**DOI:** 10.1021/acs.jpcb.5c08148

**Published:** 2026-02-27

**Authors:** Danilo Roccatano

**Affiliations:** School of Engineering and Physical Sciences, 4547 University of Lincoln, Brayford Pool, Lincoln LN6 7TS, U.K.

## Abstract

Flexible protein regions, often enriched in glycine- and serine-rich segments, play a central role in biomolecular dynamics and function. The combination of time-resolved fluorescence resonance energy transfer (FRET) spectroscopy and molecular dynamics simulations provides a powerful framework to characterize these motions at atomic resolution. In this work, we investigate the conformational and kinetic properties of Trp-(GS)_*n*_-Dbo and Trp-(PP)_*n*_-Dbo peptides (*n* = 0, 1, 2, 3) in aqueous solution using microsecond-scale MD simulations, informed by an improved description of the Dbo-labeled aspartic residue compatible with the G54A7 force field. The simulations quantitatively reproduce experimental end-to-end distances derived from FRET measurements, with deviations below 5% for all (GS)_*n*_ peptides, and correctly capture the systematic relationship between chain flexibility and fluorophore separation. Analysis of looping kinetics further shows quantitative agreement with experimentally measured contact formation rates after viscosity correction, supporting a diffusion-controlled mechanism for intrachain contact formation. Together, these results establish a consistent, quantitative link between structural ensembles, dynamical observables, and FRET experiments, and provide benchmark data for modeling fluorophore-labeled peptides and intrinsically disordered protein segments.

## Graphical abstract

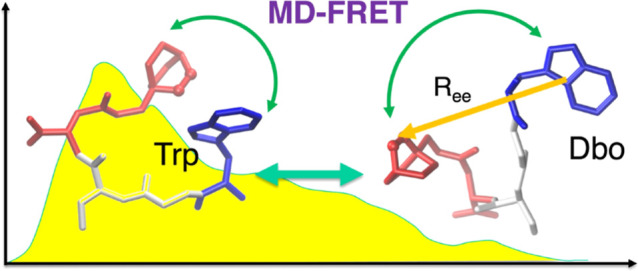

## Introduction

Short peptides provide valuable model systems for probing the conformational dynamics of intrinsically disordered protein regions, such as loops and linkers.^1^ Their reduced complexity enables detailed comparisons between molecular dynamics (MD) simulations^2^ and single-molecule spectroscopic techniques, particularly Förster resonance energy transfer (FRET).^3–6^ In such studies, the choice of fluorophores and the accuracy of the underlying force fields are crucial for establishing reliable structure–dynamics relationships. Among several donor–acceptor pairs, the intrinsic fluorophore tryptophan combined with the extrinsic probe 2,3-diazabicyclo[2.2.2]­oct-2-ene (DBO) has proven especially useful for characterizing peptides with end-to-end distances in the nanometer range.^7–9^ This pair has been widely applied to study the flexibility of short protein sequences.^10–13^ In particular, glycine/serine-rich sequences^8,13–17^ and polyproline peptides^18–20^ have been investigated in detail, as they serve as reference systems for random coil conformations in aqueous solution. Glycine- and serine-rich fragments, which are common in flexible protein regions, are particularly suitable for exploring how sequence composition influences conformational dynamics.^21–23^ In earlier work, we developed a GROMOS43A1 (G43A1)^24^ based force-field model of a modified Aspartic residue linked to the DBO probe and benchmarked MD simulations against experimental FRET data.^8,12,13,15,18^ The results showed a good agreement between simulated and FRET-derived end-to-end distances and orientational factors, validating the model. The GS-based peptides predominantly adopted compact, disordered states consistent with polypeptides in poor solvents,^8,13,15^ while the polyproline consistently reproduced the expected extended state.^18–20^

This study provided key benchmarks for understanding the conformational and dynamical behavior of short peptides in solution and for quantitatively linking FRET experiments with molecular simulations. Building on this work, the present study extends the analysis to Trp-(GS)_*n*_-Dbo peptides (*n* = 0, 1, 2, 3) and to polyproline peptides of equivalent length. While glycine/serine sequences represent highly flexible, coil-like chains, polyprolines serve as comparatively rigid, rod-like reference systems. This contrast enables a systematic investigation of how sequence-dependent flexibility influences both equilibrium conformational sampling and intramolecular dynamics probed by FRET experiments. Although previous studies demonstrated the feasibility of combining time-resolved FRET measurements with MD simulations, they were largely limited to earlier generations of force-field descriptions. Recent developments in the GROMOS force field, culminating in the G54A7 parameter set with refined backbone and nonbonded interactions, provide a more balanced and physically consistent description of peptide flexibility and intramolecular interactions.^25^ In this context, an improved representation of the Dbo-labeled aspartic residue compatible with G54A7 enables a direct and quantitative comparison between simulation results and experimental observables. The present work combines an improved, G54A7-consistent description of the Dbo-labeled aspartic residue with extensive MD simulations to investigate the structural and dynamical properties of fluorophore-labeled peptides. By comparing flexible glycine/serine-rich chains with rigid polyproline analogues, we assess how sequence-dependent flexibility is reflected in conformational sampling, end-to-end distance distributions, and intrachain dynamics probed by FRET experiments. The simulations reproduce experimental end-to-end distances with high accuracy and capture the systematic relationship between chain flexibility and fluorophore separation. Analysis of looping and contact-formation kinetics further yields quantitative agreement with experimentally measured rates after viscosity correction, consistent with a diffusion-controlled mechanism for intrachain contact formation on experimentally relevant time scales. Through microsecond-scale MD simulations, we characterize the structural ensembles and dynamical behavior of these systems, providing physical insight into how sequence composition governs both conformational sampling and time-dependent behavior in solution, and establishing quantitative benchmarks for the interpretation of FRET experiments on fluorophore-labeled peptides and intrinsically disordered protein segments.

## Methods

### Fluorophore Force Field and MD Simulations

The C-terminal Dbo residue is derived from an Asp amino acid, in which the side-chain carboxylate is replaced by an amide linkage to an amino-functionalized DBO fluorophore.^8,13^ In the present work, the G43A1-based model^13^ was updated to ensure full compatibility with the G54A7 force field,^25,26^ The complete set of revised Dbo parameters is reported in Tables S1–S4 of the Supporting Information. Peptides with sequences Trp–(GS)_*n*_–Dbo and Trp–(PP)_*n*_–Dbo (*n* = 0–3) were simulated using the SPC water model.^27^ This water model was chosen to preserve the internal consistency of the GROMOS force field. While SPC underestimates the experimental viscosity of water, this limitation primarily affects dynamical time scales and can be addressed through a posteriori viscosity-based corrections, as described below. All peptides were built in an extended conformation. Following the experimental conditions,^10^ the simulations were performed at pH 7; thus, the N-terminus is protonated, and the C-terminus is amidated, giving each peptide a net charge of +1. Each peptide was solvated in a periodic cubic box of side length 5.0 nm, removing water molecules closer than 0.15 nm to any peptide atom. A single chloride ion was added to neutralize the total charge. Table 1 provides a list of all simulated systems along with the total number of water molecules. All systems were energy-minimized using the steepest descent algorithm for at least 500 steps. Temperature was maintained at 300 K using weak coupling (coupling time τ_*T*_ = 0.1 ps), applied separately to the peptide and solvent.^28^ Pressure was controlled at 1 bar using the Berendsen barostat (τ_*P*_ = 0.5 ps).^28^ All covalent bonds were constrained using the LINCS algorithm,^29^ and water geometry was constrained using SETTLE.^30^ Simulations were performed using the GROMACS 2023 package,^31,32^ employing the time-reversible Verlet integrator. A single cutoff of 1.4 nm, as recommended for the GROMOS force field when used with the time-reversible Verlet scheme implemented in GROMACS,^33^ was applied to both Lennard–Jones and Coulomb interactions, and the neighbor list was updated every 20 integration steps. Electrostatics were treated using the generalized reaction field method with a reaction-field dielectric constant of 61,^34^ while the dielectric permittivity within the cutoff was set to 1, in accordance with standard GROMOS practice. The integration time step was 2 fs, and initial velocities were drawn from a Maxwell–Boltzmann distribution at 300 K. All systems were first equilibrated with positional restraints applied to the peptide to allow solvent relaxation, after which unrestrained production simulations of 1 μs were performed.

### Analyses of MD Trajectories

Shear Viscosity of the SPC Water Model. The shear viscosity of the SPC water model was determined using nonequilibrium MD (NEMD) simulations following the method of Hess.^35^ A spatially varying external acceleration field of the form $a_{z}\left(x\right)=A\;\cos\left(2\pi x/L_{x}\right)$ was applied along the *z*-direction, with amplitude *A* = 0.025 nm ps^–2^. This perturbation induces a stationary velocity profile *v*_*z*_(*x*), from which the shear viscosity η can be extracted analytically.^35^ The velocity profiles were analyzed to compute the instantaneous inverse viscosity. The average inverse viscosity over the production trajectory was $\overset{®}{\left(1/\eta \right)}=2245.61mskg^{-1}$ , which corresponds to a shear viscosity of $\eta =\frac{1}{\overset{®}{\left(1/\eta \right)}}=\left(0.445\pm 0.005\right)mPa\;s$ The statistical uncertainty was evaluated via block averaging of the inverse-viscosity time series.^36^ This value, in excellent agreement with previous NEMD determinations,^35,37^ was used to rescale the end-to-end contact time (see below).

Analysis of the Peptide Conformations. The peptide secondary structure was determined using the DSSP algorithm of Kabsch and Sander.^38^ The solvent-accessible surface area (SASA) and the radius of gyration (*R*_*g*_) were further computed to provide a qualitative description of the overall folding landscape of each peptide in solution. SASA values were obtained using the algorithm of Eisenhaber et al.^39^

Cluster analysis of the trajectories was performed following the method of Daura et al.,^40^ using 10,000 conformations sampled at 100 ps intervals from each peptide simulation. Structural similarity between conformations was assessed using the positional root-mean-square deviation (RMSD), and clusters were constructed iteratively by identifying the structure with the largest number of neighbors within a cutoff radius, assigning its cluster, and removing its members from further consideration. All peptide non-hydrogen atoms were used in the analysis. The clustering cutoff was selected between 0.2 and 0.35 nm (see Table S5 in the Supporting Information) to produce a comparable number of clusters across all systems. Statistical uncertainties for all average quantities were estimated using the block-averaging method.^41^

End-to-End Distance and Orientational Factor. To enable comparison with experimental FRET measurements, we extracted both the end-to-end distance (*R*_*ee*_) and the orientational factor (κ^2^).^4^ The end-to-end distance was defined as the distance between the center of the Trp indole fusion bond and the center of the diazo group of the Dbo residue (Figure 1). The probability distribution of *R*_*ee*_ was modeled using a sum of three skewed Gaussian functions

1 $$P\left(R_{ee};A_{j},r_{j}^{0},\sigma_{j}\right)=\sum_{j=1}^{3}A_{j}R^{2}\;\exp\left[-\frac{1}{2}{\left(\frac{R_{ee}-r_{j}^{0}}{\sigma_{j}}\right)}^{2}\right]$$

where *A*_*j*_, r_*j*_^0^, and σ_*j*_ are fitting parameters. This functional form represents a simple approximation of polymer end-to-end distance statistics, and it is commonly used to model FRET-derived distance distributions of flexible peptides.^3,23,42^ In our analysis, three Gaussian terms (nine parameters) were required to adequately reproduce the simulation-derived *R*_ee_ distributions. Reducing the model to two Gaussians resulted in visibly poorer fits, particularly in the short-distance region. This region is especially important for comparison with FRET experiments, as it captures donor–acceptor contact configurations where the uncertainties associated with the orientational factor and FRET efficiency are highest.^4^

**Figure 1:**
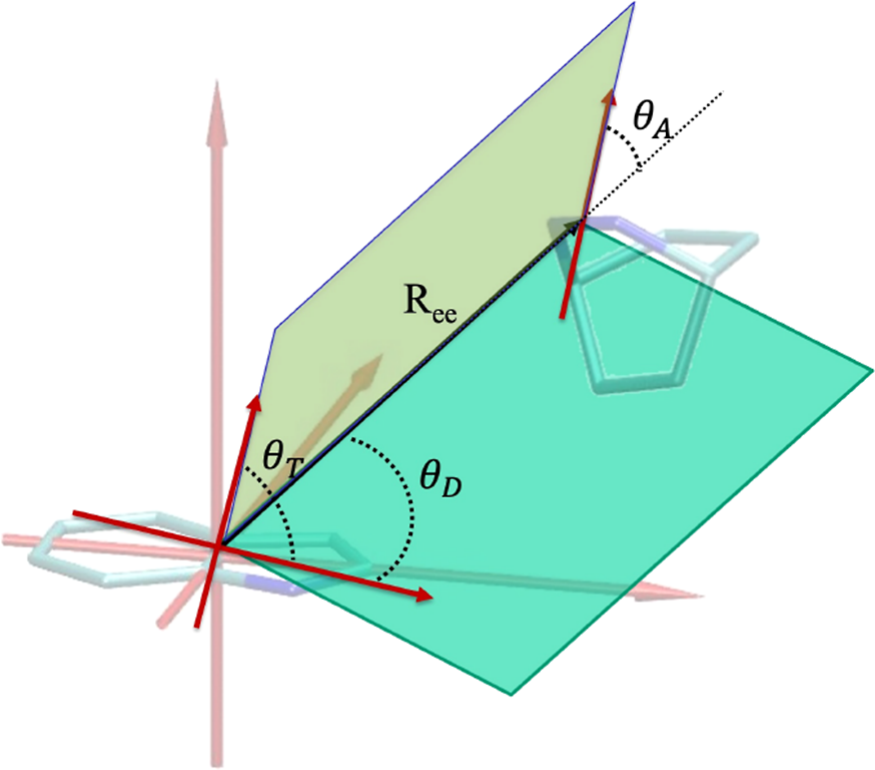
Definition of the polar angles θ_*D*_, θ_*A*_, and θ_*T*_ and the end-to-end vector ${\mathrm{R⃗}}_{ee}$ used in eq 2 for computing the orientational factor κ^2^.

The orientational factor κ^2^ was calculated from the relative orientation of the Trp and Dbo transition dipole moments using the standard Förster expression^4,43^

2 $$\kappa^{2}={\left(\sin\theta_{T}-3\;\cos\theta_{D}\;\cos\theta_{A}\right)}^{2}$$

where θ_*D*_ and θ_*A*_ are the angles between each dipole moment and ${\mathrm{R⃗}}_{ee}$ , and θ_*T*_ is the dihedral angle between the two dipoles (Figure 1).

Free energy landscape of SASA versus radius of gyration The free energy landscape in the parameter space of SASA as a function of the radius of gyration (*R*_g_) is also calculated. This representation is usually used to provide a reduced representation of the folding landscape sampled during MD simulations by peptides.^44^ The function is computed by evaluating the joint probability distribution of the two parameters on a grid of 50 × 50 points. The number of conformation occurrences at each grid point has been counted. The joint probability function was then converted into free energies using the Boltzmann inversion formula

3 $$\Delta F\left(R_{g},SASA\right)=-RT\;\ln\left(\frac{n\left(R_{g},SASA\right)}{N}\right)$$

the Δ*F*(*R*_g_, SASA) represents the change in free energy, where *R* is the gas constant, and *T* is the temperature in Kelvin, *n*(*R*_g_, SASA) is the count of conformations at a specific grid point, and *N* is the total number of analyzed conformations.

Contact Formation Kinetics. Looping rates (end-to-end collision rate constants) were calculated from the time series of the donor–acceptor separation *R*_ee_(*t*) between the Dbo and Trp chromophores. The analysis follows the first-contact-time (FCT) framework used in refs 45,46 , where a contact event is defined as the first passage of *R*_ee_(*t*) below a predefined capture distance *r*, referred to as the *sink radius*. The sink radius represents the effective spatial range within which donor–acceptor contact (and quenching) can occur, accounting for chromophore size and local conformational fluctuations. Intercontact intervals were obtained from the MD trajectory as

4 $$\Delta_{k}=\left\{t_{j}-t_{i}|R_{ee}\left(t_{i-1}\right)>r,R_{ee}\left(t_{i}\right)\leq r,R_{ee}\left(t_{j}\right)>r\right\}$$

for all *i* < *j* and *k* = 1, ..., *n*_int_. Here, *n*_int_ is the total number of independently detected looping intervals. The mean first-contact time (MFCT) from simulation was then computed using the equation

5 $$T_{sim}=\frac{{\left\langle \Delta^{2}\right\rangle }_{sim}}{2{\left\langle \Delta \right\rangle }_{sim}}$$

which gives to the mean time between two successive contact events in a diffusion-limited process.

The experimentally derived FRET kinetics were measured in D_2_O. Because the SPC water model is known to underestimate the shear viscosity of experimental heavy water,^17,37,47,48^ a direct comparison of simulated and experimental kinetic observables requires a viscosity correction. Under the assumption that diffusive conformational kinetics scale linearly with solvent viscosity, as predicted by Kramers-type theories and commonly applied in peptide looping and contact-formation studies,^17^ all simulated mean first-contact times (MFCTs) and correlation times were rescaled according to

6 $$T_{sim}^{corr}=T_{sim}\times \frac{\eta_{D_{2}O}}{\eta_{SPC}}$$

here, η_SPC_ = 0.445 mPa·s is the shear viscosity of SPC water determined from independent nonequilibrium MD simulations (see below), and $\eta_{D_{2}O}=1.10$ mPa·s is the experimental viscosity of heavy water at ambient conditions.^48^ This yields a viscosity correction factor

$$f=\frac{\eta_{D_{2}O}}{\eta_{SPC}}=2.47$$

which was applied uniformly to all peptides. Equilibrium structural properties were not affected by this rescaling.

All kinetic analyses were performed using custom Python scripts implementing a first-contact-time detection algorithm. Trajectories were prefiltered using a Savitzky–Golay smoothing routine^49,50^ to reduce high-frequency noise prior to FCT detection. For each peptide, the optimal sink radius *r* was determined by minimizing the deviation between corrected MFCTs and their experimental counterparts. Statistical uncertainties were estimated from the variance of the independent intercontact intervals, and agreement with experimental data was assessed by comparing propagated simulation errors with experimental uncertainties.

Time Series Analysis The dynamical behavior of each peptide was also characterized through time series analysis of two observables: the *R*_ee_ distance, and the Cα–Cα distance (*R*_CαCα_) between the terminal residues. All analyses were performed using custom Python scripts based on the NumPy and SciPy scientific libraries.^50,51^

### Autocorrelation and Correlation Times

The normalized autocorrelation function (ACF) was computed using a fast Fourier transform (FFT) estimator

7 $$C\left(\tau \right)=\frac{\left\langle x\left(t\right)x\left(t+\tau \right)\right\rangle -\langle x{\left(t\right)\rangle }^{2}}{\left\langle x^{2}\left(t\right)\right\rangle -\langle x{\left(t\right)\rangle }^{2}}$$

where *x*(*t*) denotes either *R*_ee_ or *R*_CαCα_. The integrated correlation time was evaluated as

8 $$\tau_{corr}=\int_{0}^{\tau_{0}}C\left(\tau \right)d\tau$$

with τ_0_ defined as the first zero crossing of the ACF.^52^ Cross-correlations between *R*_ee_ and *R*_CαCα_ were also computed to quantify how chain extension is dynamically coupled to terminal backbone separation.^12^

### Hurst Exponent

To characterize temporal correlations and long-range memory effects in peptide conformational dynamics, we evaluated the Hurst exponent *H* for both *R*_ee_ and *R*_CαCα_ time series. The Hurst exponent provides a quantitative measure of persistence in stochastic processes and allows discrimination between purely diffusive fluctuations and dynamics influenced by internal friction or conformational constraints. The exponent *H* was determined using the classical rescaled-range (R/S) analysis^53,54^

9 $$\frac{R\left(n\right)}{S\left(n\right)}=cn^{H}$$

where *R*(*n*) denotes the range of cumulative deviations and *S*(*n*) the corresponding standard deviation within a window of size *n*. Values of *H* > 0.5 indicate persistent dynamics, reflecting correlated conformational motions along a rugged free-energy landscape, whereas *H* < 0.5 corresponds to antipersistent behavior dominated by restoring forces. The limiting case *H* = 0.5 describes uncorrelated Brownian diffusion. Deviations from Brownian behavior, therefore, provide insight into conformational memory effects that influence the temporal averaging of experimentally derived observables.

### Power Spectral Density

To complement the time-domain analysis, the power spectral density (PSD) of the same time series was computed using Welch’s method,^55^ employing Hanning windows with 50% overlap. Spectral analysis enables separation of dynamical contributions occurring on different time scales and provides insight into the hierarchy of motions sampled by the peptides.

The high-frequency region of the PSD was fitted to a power-law form

10 $$S\left(f\right)\propto f^{-\alpha }$$

yielding the spectral exponent α. Different values of α correspond to distinct noise regimes: α ≈ 0 indicates white noise dominated by fast, uncorrelated local motions; α ≈ 1 corresponds to 1/*f* noise associated with scale-free dynamics and the coupling of multiple time scales; and α ≈ 2 reflects Brownian noise characteristic of slow, diffusive conformational rearrangements.

Together with the Hurst exponent, the PSD analysis provides a consistent description of peptide dynamics across time and frequency domains, linking fast local fluctuations to slower global conformational changes and clarifying their impact on experimentally measured, time-averaged FRET observables.

## Results and Discussion

### General Structural Features

The DSSP analysis revealed that all peptides are predominantly disordered, with coil structures representing the major secondary-structure component across the series. Only the polyproline peptides displayed appreciable β-strand content, increasing systematically with peptide length and reaching 57% for (PP)_3_. In contrast, (GS)_3_ exhibited the highest loop content (55%), consistent with the expected conformational flexibility of Gly/Ser-rich sequences.

We also assessed conformational diversity using the clustering approach described in the Methods section. Figure 2 shows the cumulative number of identified clusters as a function of simulation time. In all cases, the curves exhibit clear plateaus, indicating satisfactory sampling of the conformational landscape.^56^ The cluster analysis revealed a marked contrast between the flexible GS peptides and the more structurally restricted polyproline series. For (GS)_2_ and (GS)_3_, the three most populated clusters account for approximately 50% of all sampled conformations, as quantified explicitly in Table S5 of the Supporting Information and illustrated by the cumulative population curves in Figure 2. This distribution reflects a broad and diverse conformational ensemble, consistent with the expectation that short disordered peptides explore a rugged and heterogeneous energy landscape.^57^ In contrast, the polyproline peptides exhibit a much narrower distribution, with the three dominant clusters accounting for more than 70% of the population, as shown in Figure 2 and reported numerically in Table S5 of the Supporting Information.

**Figure 2:**
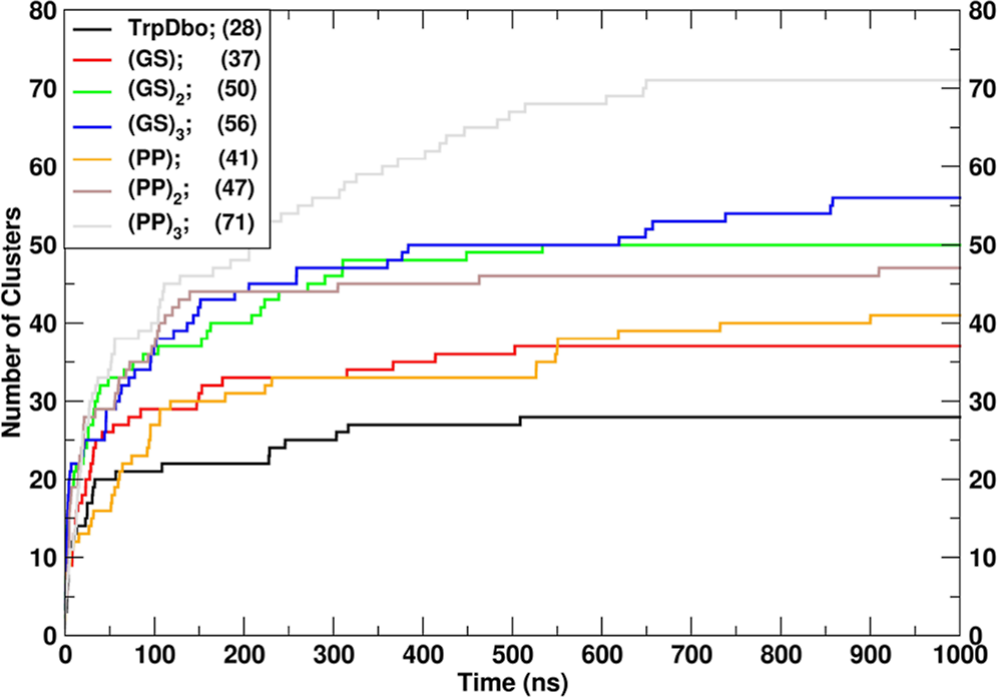
Cumulative number of clusters as a function of simulation time.

**Table 1:** Summary of Peptide Simulations

name	sequence	water molecules
TrpDbo	Trp-Dbo-NH_2_	2248
(GS)	Trp-(GlySer)-Dbo-NH_2_	2241
(GS)_2_	Trp-(GlySer)_2_-Dbo-NH_2_	2144
(GS)_3_	Trp-(GlySer)_3_-Dbo-NH_2_	3119
(PP)	Trp-(ProPro)-Dbo-NH_2_	2237
(PP)_2_	Trp-(ProPro)_2_-Dbo-NH_2_	2135
(PP)_3_	Trp-(ProPro)_3_-Dbo-NH_2_	3114

Representative structures of these dominant clusters are shown in Figure 3. As expected, the GS peptides sample compact conformations due to the increased flexibility imparted by glycine, whereas the polyproline peptides maintain extended architectures dictated by the rigidity of the proline backbone. These trends are consistent with *R*_g_ reported in Table 2. Across the series, *R*_g_ increases monotonically with peptide length, spanning 0.46–0.80 nm. The polyproline peptides, particularly for *n* ≥ 2, exhibit larger *R*_g_ and SASA values than their GS analogues, reflecting their more extended conformations. Table S2 in the Supporting Information provides full cluster statistics including ⟨*R*_g_⟩, ⟨*R*_ee_⟩ and the population relative contributions (PRC) of the three most populated clusters. The GS peptides populate several compact conformational states with similar *R*_g_ values and moderately distributed cluster populations (27–37% for the most populated cluster). By contrast, the polyproline peptides occupy more extended and conformationally restricted states, characterized by larger *R*_g_ values and a clearly dominant first cluster (45–57% for PP, PP_2_, and PP_3_).

**Figure 3:**
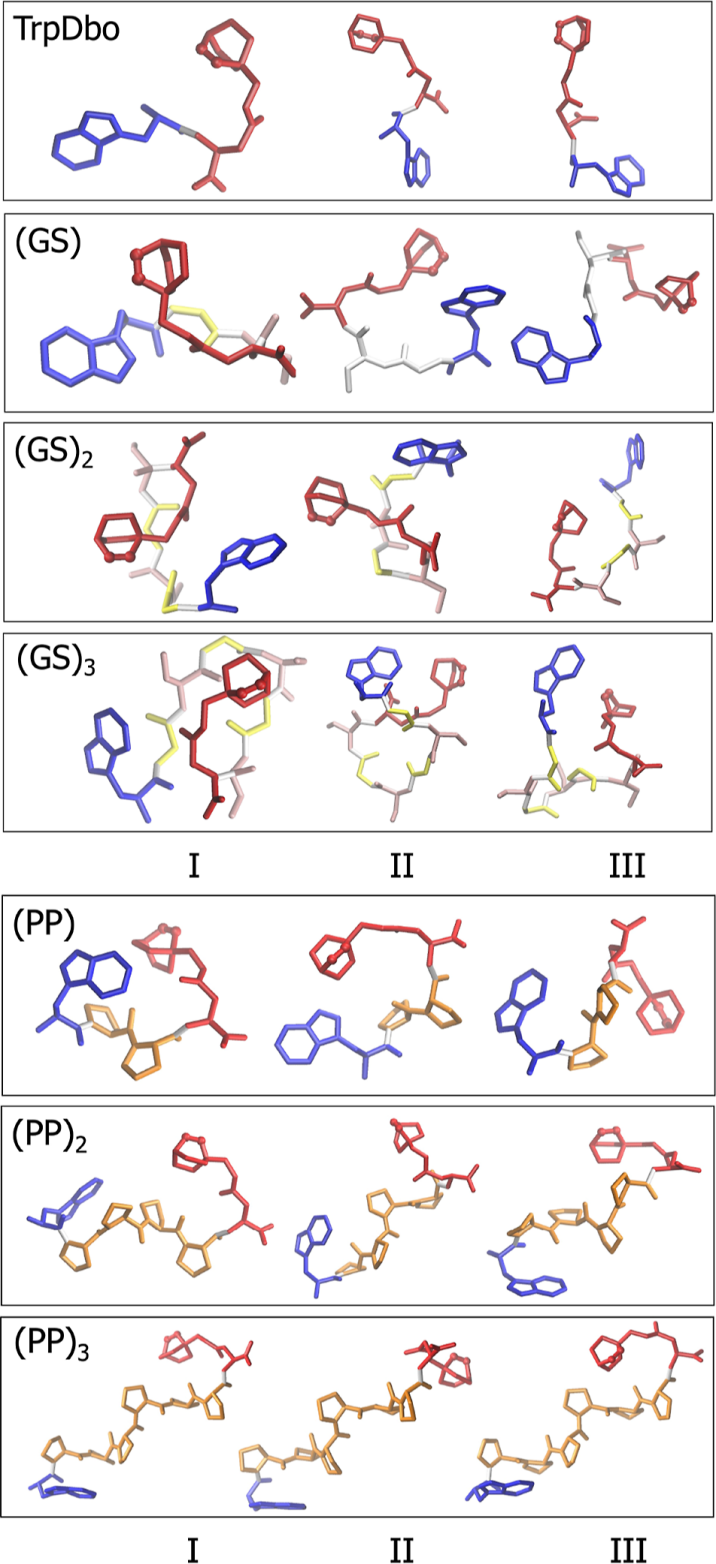
Representative structures from the first three clusters of each simulation. Glycine residues are shown in cyan, serine in pink, proline in orange, Dbo in red (nitrogen atoms as spheres), and tryptophan in blue.

**Table 2:** Average Structural Parameters for all Peptides^a^

peptide	*R*_g_ (nm)	SASA (nm^2^)	% loop	% PP	% coil
trpDbo	0.463 (0.001)	6.61 (0.01)	–	–	100
(GS)	0.507 (0.002)	8.06 (0.01)	26	1	73
(GS)_2_	0.537 (0.002)	8.80 (0.02)	45	–	55
(GS)_3_	0.567 (0.004)	9.87 (0.04)	55	–	45
(PP)	0.481 (0.001)	8.10 (0.01)	25	12	63
(PP)_2_	0.618 (0.003)	10.23 (0.02)	17	46	37
(PP)_3_	0.797 (0.002)	12.60 (0.02)	13	57	30

a For each simulation, the radius of Gyration (*R*_g_), the solvent-accessible surface area (SASA), and the percentages of secondary-structure content calculated with the DSSP method are reported. The loop content corresponds to the sum of turn and bend Structures. Standard deviations are shown in parentheses

Figure 4 shows the contour plots of the free energy landscapes Δ*F*(*R*_g_, SASA) for the (GS)_*n*_ and (PP)_*n*_ peptide sequences. The resulting potentials of mean force (PMFs) display the characteristic comet-shaped profile previously observed for the (GS)_3_, (GG)_3_, and (SS)_3_ peptides.^15^ For the (GS) peptides, the position of the free-energy minimum reflects a preference for compact, disordered conformations and is located at relatively small values of the radius of gyration *R*_g_. In contrast, the more rigid polyproline peptides exhibit a systematic shift of the free-energy minimum toward larger *R*_g_ values as the chain length increases, consistent with their intrinsically extended backbone geometry.

**Figure 4:**
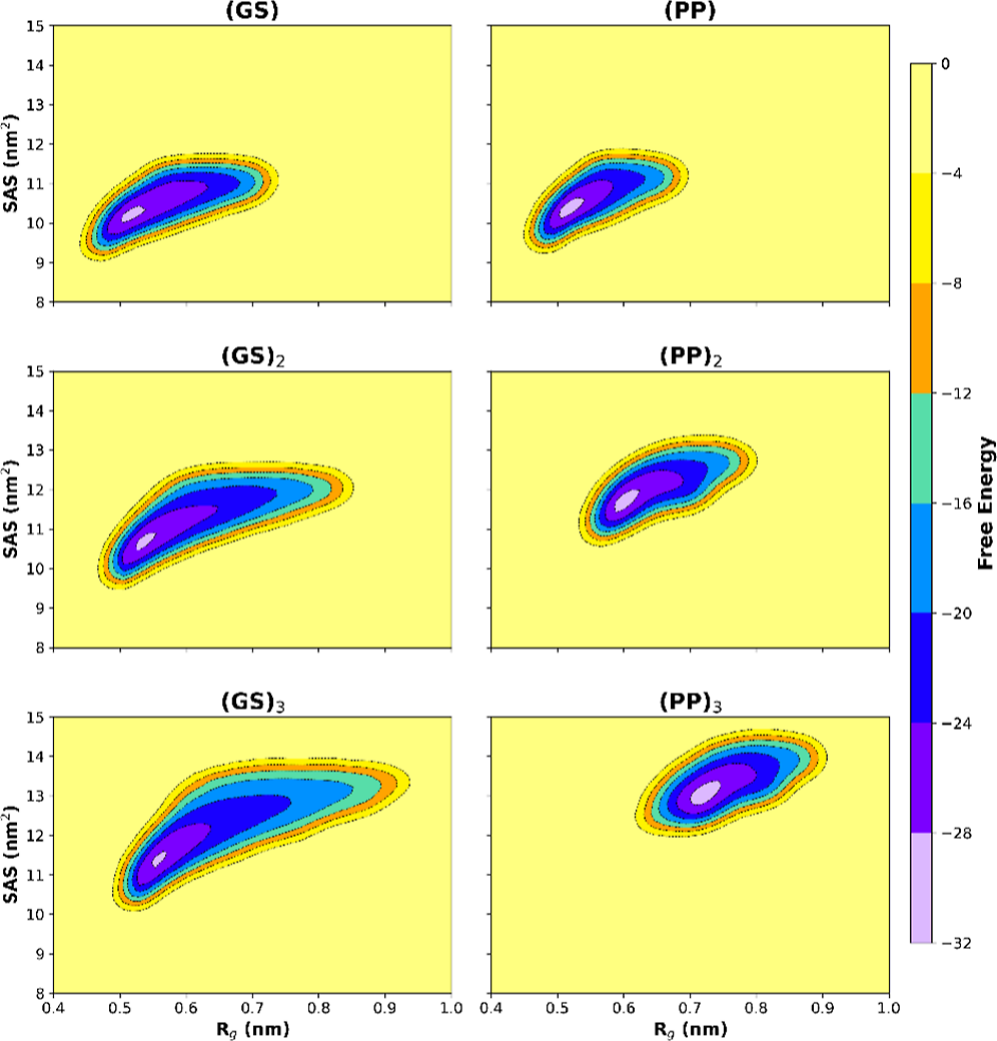
Contour plots of the free energy landscapes Δ*F*(*R*_g_, SASA) derived from simulations of the (GS)_*n*_ and (PP)_*n*_ peptides.

### End-to-End Distance Distributions and Orientation Factor

Figures 5 and 6 illustrate the *R*_ee_ distance distributions for all peptides. Each figure presents the distributions as gray silhouettes overlaid with multi-Gaussian fits (orange lines). The colored curves correspond to the distributions obtained from the first three conformational clusters, scaled by their relative populations, with their sum shown as dashed red lines. All distributions are multimodal, featuring a main peak at short distances and, depending on the peptide, additional smaller peaks. The positions of these features coincide with the main peaks observed in the first three cluster distributions.

**Figure 5:**
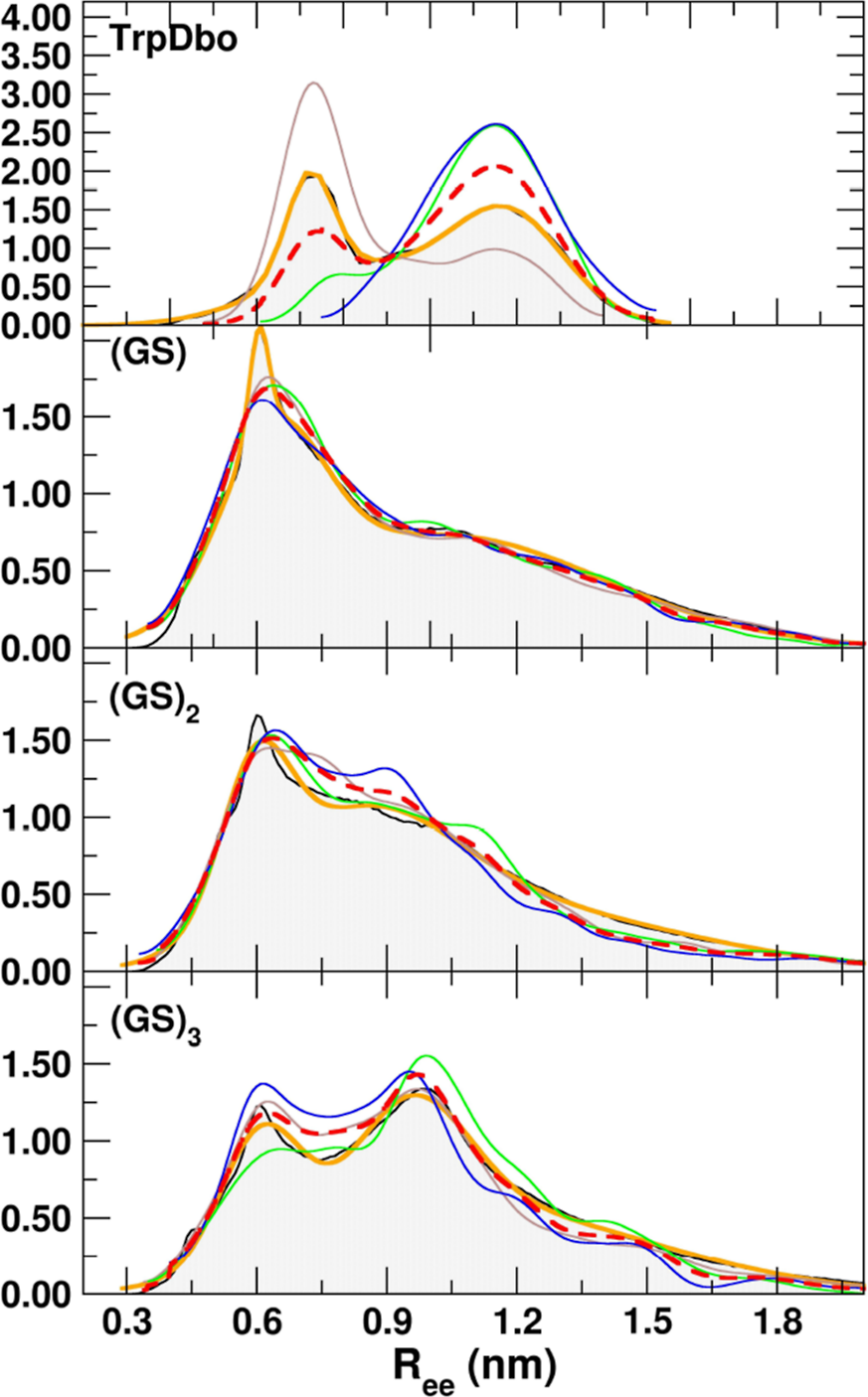
Distribution of *R*_ee_ distances for all conformations of the (GS)_*n*_ peptides (gray silhouette) with multi-Gaussian fits (orange line). Cluster-specific distributions are shown in brown, green, and blue, scaled by their relative populations (see Table S2 in the Supporting Information).

**Figure 6:**
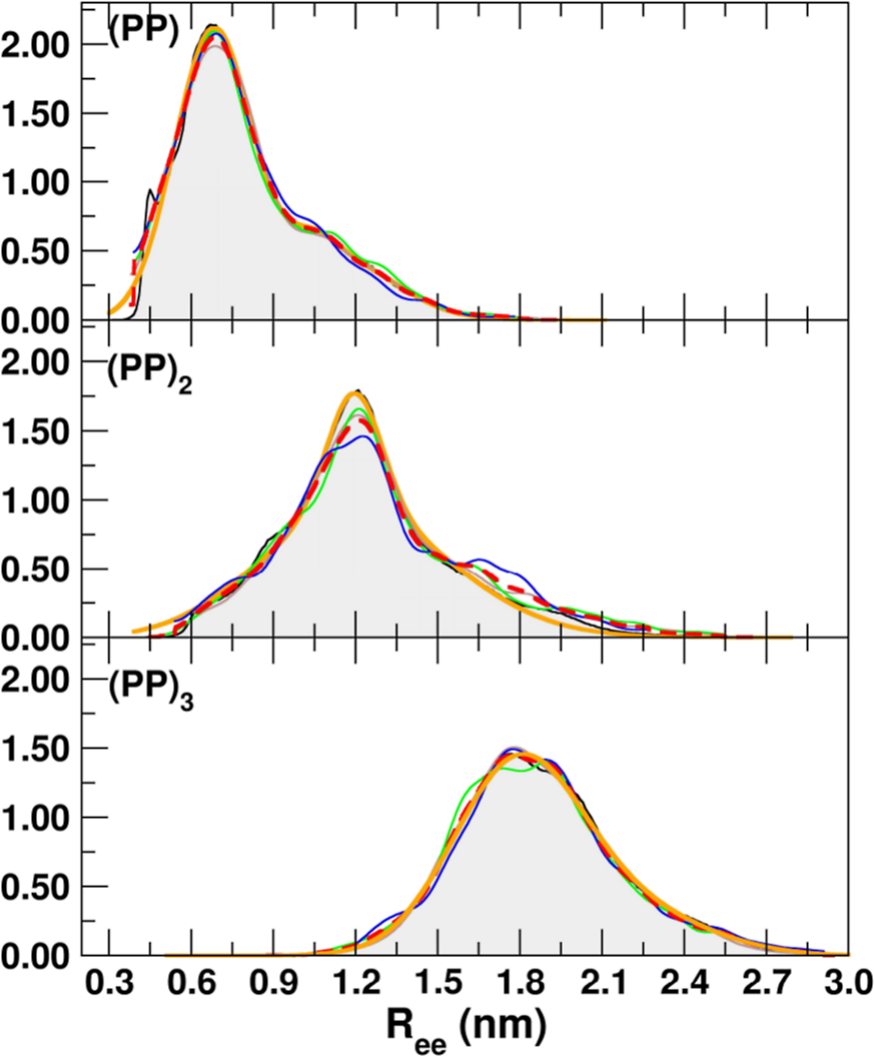
As in Figure 5, but for the (PP)_*n*_ peptides.

For TrpDbo and (GS)_3_, two distinct peaks at short and longer distances are clearly visible. For the other (GS) peptides, the peaks at longer distances overlap and are less distinct. The distributions were fitted using triple-exponential functions (see Methods), which provided excellent representations of the data for the GS series, as also noted in previous studies.^13,15,18^ In contrast, the extended conformations of the polyproline peptides yield distributions with a single dominant peak. Because of this characteristic, even a single exponential function can adequately describe their profiles.

The average *R*_ee_ distances obtained from the simulations are compared in Table 3 with those reported in previous works using the G43A1 version of the force field^13,15^ and with the values estimated from experimental data. The comparison highlights the improved performance of the updated G54A7-based model relative to the earlier G43A1 description. For the GS series, the *R*_ee_ values predicted with G54A7 (0.92–1.02 nm) are in excellent agreement with the experimental data (0.90–1.01 nm), whereas the G43A1 results (0.80–0.88 nm) systematically underestimate chain extension. This improvement is particularly evident for (GS)_3_, where the G43A1 prediction of 0.88 nm falls below the experimental value (1.01 nm), while G54A7 reproduces it almost quantitatively (1.02 nm). This trend indicates that the refined parameter set provides a more realistic balance of backbone flexibility and fluorophore interactions in disordered peptide ensembles. For the PP peptides, which are intrinsically more rigid, the differences between the two force fields are smaller. In addition to the donor–acceptor distance, Table 3 also reports the terminal Cα–Cα distance *R*_CαCα_, which provides a direct measure of backbone end-to-end extension, independent of fluorophore size, linker flexibility, or orientational effects. This quantity serves as a structural reference for interpreting the fluorescence-based *R*_ee_ values. For the GS peptides, *R*_CαCα_ remains relatively small (0.82–0.87 nm) and shows only a weak dependence on chain length, consistent with their compact and highly flexible disordered conformations. In contrast, the polyproline peptides exhibit a pronounced and systematic increase in *R*_CαCα_ with chain length, from 0.91 nm for (PP) to 2.10 nm for (PP)_3_, reflecting the stiffness of the proline backbone and the tendency to form extended, rod-like conformations. The TrpDbo peptide displays the smallest Cα–Cα separation (0.38 nm), indicative of a highly compact backbone arrangement. Nonetheless, GROMOS54A7 generally produces values closer to the experiment. For example, the *R*_ee_ for (PP)_2_ improved from 1.13 nm (G43A1) to 1.24 nm (G54A7), in excellent agreement with the experimental estimate of 1.23 nm. The longest proline chain, (PP)_3_, was slightly overextended in G54A7 (1.89 nm versus 1.66 nm), while G43A1 slightly underestimated it (1.80 nm). Despite this, both force fields reproduced the overall rigidity and extended nature of proline-rich chains.

**Table 3:** Calculated and Experimental Parameters for all Peptides^a^

peptide	*R*_CαCα_^G54A7^ (nm)	*R*_ee_^G54A7^ (nm)	*R*_ee_^GR96^ (nm)	*R*_exp_ (nm)	⟨κ^2^⟩
trpDbo	0.38	0.97	0.86	0.87	0.589
(GS)	0.84	0.92	0.80	0.90	0.631
(GS)_2_	0.87	0.95	0.82	0.97	0.673
(GS)_3_	0.82	1.02	0.88	1.01	0.619
(PP)	0.91	0.81	0.89	0.82	0.673
(PP)_2_	1.53	1.24	1.13	1.23	0.791
(PP)_3_	2.10	1.89	1.80	1.66	0.686

a The calculated uncertainties are σ_*R*_ee__ = 0.01 nm and $\sigma_{\left\langle \kappa^{2}\right\rangle }=0.005$ . Experimental end-to-end distances *R*_exp_ are taken from published Trp–DBO FRET measurements^8,13,18^ and were obtained by converting FRET efficiencies into distances using the Förster formalism. Experimental values have an estimated error of about 5%.

The average orientation factor (κ^2^) distributions further support these structural trends. The GS peptides exhibit relatively broad κ^2^ distributions, consistent with the greater orientational freedom of the Trp-DBO donor–acceptor pair, whereas the PP peptides display narrower profiles reflecting their more restricted conformational sampling. The mean values of ⟨κ^2^⟩ (Table 3) fall within the range expected for flexible versus conformationally constrained peptide chains. To assess the donor–acceptor relative orientation more quantitatively, we compared the average ⟨κ^2^⟩ values for all peptides. Most peptides exhibit mean orientation factors close to the isotropic limit of 2/3, with deviations of typically 5–10%. The polyproline dimer (PP_2_), which samples particularly restricted backbone geometries, shows a larger deviation (approximately 0.79), consistent with its more rigid conformational landscape.

Overall, these results demonstrate that the refined DBO residue parameters, combined with the GROMOS54A7 backbone description, provide a substantially improved representation of peptide end-to-end distances. The excellent agreement with the FRET benchmarks (particularly for the GS series) underscores the reliability of the updated model for quantitative spectroscopic comparisons.

### Time Series Analysis

We also analyzed the *R*_ee_ and *R*_CαCα_ time series using Hurst exponents, autocorrelation times, PSD scaling, and the zero-lag cross-correlation between the two observables. The resulting dynamical indicators are summarized in Table 4, allowing a direct comparison of the dynamical behavior of global chain extension and backbone end-to-end fluctuations.

**Table 4:** Summary of the Main Dynamical Indicators Extracted from Time-Series Analysis of the Peptide Simulations^a^

peptide	observable	τ_corr_ (ps)	*H*	α	noise type	cross-corr.
TrpDbo	*R*_ee_	3820.9	0.803	1.308	1/f	
	*R*_CαCα_	10.5	0.994	0.029	white	–0.888
(GS)	*R*_ee_	10722.6	0.781	1.335	1/f	
	*R*_CαCα_	1148.3	0.863	1.144	1/f	1.000
(GS)_2_	*R*_ee_	6744.8	0.762	1.326	1/f	
	*R*_CαCα_	2194.7	0.781	1.306	1/f	1.000
(GS)_3_	*R*_ee_	11071.5	0.750	1.352	1/f	
	*R*_CαCα_	2905.1	0.753	1.299	1/f	1.000
(PP)	*R*_ee_	3647.8	0.864	1.153	1/f	
	*R*_CαCα_	228.7	0.951	0.848	1/f	–0.346
(PP)_2_	*R*_ee_	47841.7	0.834	1.196	1/f	
	*R*_CαCα_	136.6	0.968	0.737	1/f	0.002
(PP)_3_	*R*_ee_	9910.9	0.856	1.160	1/f	
	*R*_CαCα_	120.8	0.956	0.898	1/f	–0.423

a *R*_ee_ is the donor–acceptor FRET distance, and *R*_CαCα_ is the terminal Cα–C*α* distance. For each observable, the table reports the integrated autocorrelation time *τ*_corr_, the Hurst exponent *H*, the PSD spectral slope α, and the corresponding noise classification. The last column reports the zero-lag cross-correlation coefficient between *R*_ee_ and *R*_CαCα_

The correlation times τ_corr_ (third column of Table 4) span a wide range, from tens of picoseconds to tens of nanoseconds. For the GS series, both observables display correlation times of comparable order of magnitude, with systematically shorter relaxation times for *R*_CαCα_, consistent with concerted backbone motions. In contrast, for Trp–Dbo and the polyproline peptides, *R*_CαCα_ relaxes significantly faster than *R*_ee_, indicating rapid local fluctuations superimposed on slower global rearrangements.

The estimated Hurst exponents *H* (fourth column of Table 4) are consistently high (*H* ≳ 0.75) for both *R*_ee_ and *R*_CαCα_ across all peptides, indicating strong long-range persistence and highly correlated dynamics. Such values are characteristic of fractional Brownian motion with pronounced memory effects^58^ and are often associated with rugged and multiscale biomolecular free-energy landscapes.^57^ Notably, the GS peptides exhibit similar *H* values for both observables, suggesting that backbone fluctuations and global chain extension are governed by the same underlying correlated motion. In contrast, the polyproline peptides show systematically higher *H* values for *R*_CαCα_ than for *R*_ee_, reflecting the enhanced rigidity of the proline backbone.

The spectral exponents α of the PSD (fifth column of Table 4) reveal predominantly 1/*f* (pink) noise behavior for *R*_ee_, indicative of scale-free dynamics. In contrast, *R*_CαCα_ exhibits a broader range of spectral exponents, with values closer to white or weakly correlated noise for Trp–Dbo and the polyproline peptides. This reflects the presence of fast, less correlated local backbone fluctuations in more rigid sequences.

Finally, the cross-correlation coefficients reported in the last column of Table 4 quantify the degree of coupling between *R*_ee_ and *R*_CαCα_. The GS peptides show strong positive correlations, indicating a concerted increase or decrease of both distances. In contrast, Trp–Dbo and the longer polyproline chains exhibit weak or negative correlations, revealing a partial decoupling between local backbone motions and global chain extension.

### End-to-End Contact Kinetics

The kinetics of donor–acceptor contact in the peptide series were analyzed using the MFCT. This is defined as the average time it takes for the donor–acceptor distance *R*_ee_(*t*) to first fall below a specified contact radius *r* (see Methods). Starting from the reference value of 0.45 nm employed in previous studies,^13^ the optimized radii (*r*_opt_) fall between 0.44 and 0.50 nm (Table 5), consistent with the expected spatial proximity of the Dbo and Trp chromophores and in line with our earlier work.^9,12,13^ To compare simulations with experimental MFCTs measured in D_2_O, all simulated times were multiplied by a viscosity correction factor of *f* = 2.472. This correction reduces the mean deviation between simulation and experiment from approximately 60% to about 4%, corresponding to an absolute reduction of roughly 56–57% across the peptide set. Statistical uncertainties in the uncorrected MFCTs (0.48–1.34 ns, or 6–15%) propagate to 1.2–3.3 ns (5–15%) after the correction, remaining well within the experimental error of approximately 3%. For all peptides, the corrected MFCTs agree with experiment within the combined uncertainty bounds, with (GS)_3_ showing marginal but still acceptable overlap due to its larger statistical uncertainty (Table 5). The optimized sink radii reflect subtle variations in chromophore accessibility and local backbone flexibility. The glycine–serine peptides display the fastest looping kinetics, consistent with their highly flexible and compact conformational ensemble. In contrast, the polyproline series reported previously,^8^ which adopt more extended and conformationally restricted structures, display progressively slower contact formation times. The strong overall agreement between the corrected MFCTs and experimental values supports a diffusion-controlled mechanism for end-to-end contact formation, consistent with theoretical expectations for disordered peptides.^12,13,45,46^ Taken together, these results demonstrate that solvent-mediated diffusion dominates the kinetics, while peptide-specific structural effects are effectively captured by optimizing the sink radius, enabling accurate and robust kinetic predictions from MD simulations.

**Table 5:** Optimized donor–acceptor Contact Formation Times with Viscosity Correction and Propagated Uncertainties^a^

peptide	*r*_opt_ (nm)	*T*_MD_ (ns)	*T*_MD_^corr^ (ns)	*T*_exp_ (ns)	error_corr_(%)
trpDbo	0.497	8.64 ± 0.84	21.36 ± 2.08	22.0 ± 0.7	2.9
(GS)	0.441	5.11 ± 0.48	12.64 ± 1.20	13.0 ± 0.4	2.8
(GS)_2_	0.441	7.12 ± 0.86	17.60 ± 2.11	17.0 ± 0.5	3.5
(GS)_3_	0.441	8.95 ± 1.34	22.13 ± 3.32	24.0 ± 0.7	7.8

a *T*_MD_ and *T*_MD_^corr^ denote the mean first-passage contact times obtained from simulations before and after viscosity rescaling, respectively. Experimental times *T*_exp_ are taken from FRET measurements. The viscosity correction uses a scaling factor of 2.472, and experimental uncertainties are assumed to be 3%

More generally, the refined force field and the combined FRET–MD framework presented here are not limited to short model peptides but are applicable to a broad class of flexible biomolecular systems. MD simulations have been extensively used to rationalize the stability and conformational behavior of flexible and designed peptides, where subtle balances between backbone flexibility and side-chain interactions shape the accessible ensembles.^59,60^ These considerations become even more relevant for intrinsically disordered regions and short protein segments, whose functional properties are governed by heterogeneous and highly dynamic conformational landscapes. Accurately capturing such disordered ensembles remains a central challenge for biomolecular force fields, and quantitative validation against experimental observables is essential for their assessment.^61,62^ In this respect, the present work extends force-field benchmarking by combining equilibrium and kinetic FRET observables, thereby validating not only average structural properties but also intramolecular diffusion and looping dynamics. More broadly, the integration of FRET-derived information with molecular simulations has emerged as a powerful strategy for resolving heterogeneous conformational ensembles in flexible systems.^63^ The refined DBO force-field model and the analysis framework developed here provide a transferable platform for applying FRET-assisted MD to intrinsically disordered segments, flexible linkers, and small proteins undergoing conformational adaptation or binding-induced rearrangements.

## Conclusions

In this work, we conducted microsecond-scale MD simulations of Trp-(GS)_*n*_-Dbo peptides (*n* = 0–3) and their polyproline analogues using the updated G54A7 force field together with newly refined parameters for the Dbo fluorophore. The simulations reveal that Gly/Ser-rich sequences predominantly adopt compact, disordered conformations, whereas proline-based peptides maintain extended, rod-like structures, consistent with their intrinsic rigidity. The incremental extension of the Gly/Ser chains increases the mean end-to-end distance by approximately 0.1 nm per residue, in excellent agreement with FRET experiments. The refined Dbo model significantly improves the quantitative reproduction of experimental donor–acceptor distances compared to the earlier G43A1 formulation, indicating a more accurate balance between peptide flexibility and chromophore interactions. When applied to contact-formation kinetics, the combination of trajectory smoothing, optimized sink radii, and viscosity correction yields mean first-contact times that agree with experiment within 5%. This demonstrates both the precision and the physical consistency of the kinetic model.

Overall, this study validates the improved Dbo force-field description and its seamless integration with G54A7 for the quantitative simulation of time-resolved spectroscopic observables. Beyond reproducing experimental FRET data, the results establish a robust computational framework for probing conformational dynamics and intramolecular diffusion in disordered and semirigid peptide chains, offering a valuable tool for benchmarking and refining biomolecular force fields.

## Supplementary Material



## Notes

The GROMOS54A7 force-field parameters of the Dbo residue, and additional structural and kinetic analyses. It includes tables reporting cluster populations, gaussian fit parameters of the end-to-end distance distributions (PDF)
