# Supplementary material for: Quantitative Integration of FRET and Molecular Dynamics for Modeling Flexible Peptides
Source: J Phys Chem B. 2026 Feb 27;130(10):2754–63. doi: 10.1021/acs.jpcb.5c08148 (PMC12990115; doi:10.1021/acs.jpcb.5c08148)
Supplement: Supplementary file 1 [file jp5c08148_si_001.pdf]

SUPPORTING INFORMATION: Quantitative Integration of FRET and Molecular Dynamics for Modeling Flexible Peptides.

Danilo Roccatano[1]

[1]School of Engineering and Physical Science, University of Lincoln, Brayford Pool, Lincoln, LN6 7TS, United Kingdom

*Email: droccatano@lincoln.ac.uk

List of Contents

Table S1-4. GROMOS54A7 force field parameters for the Dbo residue.

Table S6. Cluster analysis

Table S7. Gaussian fit parameters for R_{ee} distributions

TABLES

Table S1: Partial charges, atom types, and selected bonded parameters used for the GROMOS 54A7 Dbo residue model. The charges are in electrostatic units.

Atom name	GROMOS 54A7 atom type	Partial charge (u.a.c.)
N	N	-0.31
H	H	0.31
CA	CH1	0.00
CB	CH2	0.00
CG	C	0.380
OD1	O	-0.38
ND2	NT	-0.28
HD2	H	0.28
CZ	CH2	-0.20
C1	C	0.50
N1	NR	-0.30
C2	CH2	0.15
C3	CH2	-0.15
C5	CH2	-0.15
C6	CH2	0.15
C4	CH1	0.47
N2	NR	-0.47
C	C	0.45

Continued on next page

Table S1 – continued from previous page

Atom name	GROMOS 54A7 atom type	Partial charge (u.a.c.)
O	O	-0.45

Table S2: Bond parameters of the DBO fluorophore.

Bond	Equilibrium bond length (nm)	Force constant (kJ mol^{-1} nm^{-2})
CZ–C1	0.152	41840
C1–C2	0.154	41840
C1–N1	0.147	41840
C1–C6	0.154	41840
C2–C3	0.154	41840
C3–C4	0.154	41840
C4–C5	0.154	41840
C4–N2	0.147	41840
C5–C6	0.154	41840
N1–N2	0.122	41840

Table S3: Bond angle parameters of the DBO fluorophore.

Bond angle	Equilibrium angle (deg)	Force constant (kJ mol^{-1} rad^{-2})
ND2–CZ–C1	112	520.
CZ–C1–C2	113	520.
CZ–C1–N1	108	520.
CZ–C1–C6	112	520.
C1–C2–C3	108	520.
C1–N1–N2	116	520.
C1–C6–C5	108	520.
C2–C3–C4	116	520.
C2–C1–N1	108	520.
C2–C1–C6	109	520.
C3–C4–C5	110	520.
C3–C4–N2	108	520.
C4–C5–C6	108	520.
C4–N2–N1	115	520.
C5–C6–C1	108	520.
C5–C4–N2	108	520.
C6–C1–N1	107	520.

Table S4: Dihedral parameters of the DBO fluorophore.

Dihedral	Equilibrium dihedral angle (deg)	Force constant (kJ mol^{-1})	Multiplicity
ND2–CZ–C1–N1	0.0	3.5	3
ND2–CZ–C1–N1	0.0	0.4	2
ND2–CZ–C1–N1	0.0	0.3	1

Table S5: Properties of the three most populated clusters for each peptide. Average $\langle R_g \rangle$ and $\langle R_{ee} \rangle$ calculated from the conformations in the first three clusters (Cluster 1-3), along with their population relative contributions (PRC, in %). All distances are reported in nanometres (nm).

Simulation	Cutoff/nm	Property	Cluster 1	Cluster 2	Cluster 3
		$\langle R_g \rangle$	0.450 ± 0.002	0.474 ± 0.005	0.487 ± 0.002
TrpDbo	0.20	$\langle R_{ee} \rangle$	0.875 ± 0.004	1.103 ± 0.004	1.138 ± 0.004
		PRC	27.7	18.4	10.3
		$\langle R_g \rangle$	0.477 ± 0.001	0.474 ± 0.001	0.511 ± 0.002
(GS)	0.25	$\langle R_{ee} \rangle$	0.912 ± 0.006	0.902 ± 0.009	0.904 ± 0.010
		PRC	37.1	13.5	12.7
		$\langle R_g \rangle$	0.497 ± 0.002	0.499 ± 0.004	0.590 ± 0.002
$(GS)_2$	0.30	$\langle R_{ee} \rangle$	0.909 ± 0.006	0.931 ± 0.008	0.898 ± 0.013
		PRC	27.5	19.5	7.4
		$\langle R_g \rangle$	0.527 ± 0.003	0.530 ± 0.002	0.538 ± 0.003
$(GG)_3$	0.35	$\langle R_{ee} \rangle$	0.804 ± 0.006	0.811 ± 0.007	0.795 ± 0.008
		PRC	34.1	12.5	7.0
		$\langle R_g \rangle$	0.455 ± 0.001	0.490 ± 0.001	0.484 ± 0.003
(PP)	0.20	$\langle R_{ee} \rangle$	1.285 ± 0.005	1.282 ± 0.009	1.270 ± 0.012
		PRC	45.8	13.7	10.3
		$\langle R_g \rangle$	0.593 ± 0.001	0.616 ± 0.001	0.621 ± 0.002
$(PP)_2$	0.20	$\langle R_{ee} \rangle$	1.865 ± 0.004	1.858 ± 0.008	1.877 ± 0.009
		PRC	57.1	17.0	14.3
		$\langle R_g \rangle$	0.774 ± 0.001	0.808 ± 0.001	0.770 ± 0.001
$(PP)_3$	0.20	$\langle R_{ee} \rangle$	0.965 ± 0.006	0.991 ± 0.009	0.945 ± 0.013
		PRC	50.4	13.2	11.0

Table S6: Parameters (A_j, r_j^0, σ_j^2) from two- and three-Gaussian fits of end-to-end distance distributions for Trp-$(GS)_n$-Dbo ($n = 0, 1, 2, 3$) and Trp-$(PP)_n$-Dbo ($n = 1, 2, 3$).

Coeff.	TrpDbo	(GS)	$(GS)_2$	$(GS)_3$	(PP)	$(PP)_2$	$(PP)_3$
A_1	1.00	0.95	0.36	0.63	0.79	0.55	0.40
r_1^0	2.63	2.91	1.39	0.89	0.61	0.19	0.09
σ_1^2	1.25	1.89	2.64	2.40	1.8×10^{-5}	0.79	0.003
A_2	1.15	0.70	0.87	0.73	0.95	1.20	1.72
r_2^0	0.71	0.59	0.69	0.92	1.18	1.31	2.00
σ_2^2	0.72	0.60	0.58	0.58	3.00	0.95	1.06
A_3	0.018	0.17	0.19	0.21	0.15	0.008	0.05
r_3^0	0.003	0.015	0.057	0.018	0.010	0.13	0.11
σ_3^2	0.046	0.0005	0.006	0.009	1.4×10^{-5}	0.068	0.003
Correlation coefficient	0.998	0.997	0.997	0.997	0.994	0.996	0.999
